# Stable and Novel Quantitative Trait Loci (QTL) Confer Narrow Root Cone Angle in an Aerobic Rice (*Oryza sativa* L.) Production System

**DOI:** 10.1186/s12284-021-00471-2

**Published:** 2021-03-07

**Authors:** Ricky Vinarao, Christopher Proud, Xiaolu Zhang, Peter Snell, Shu Fukai, Jaquie Mitchell

**Affiliations:** 1 The University of Queensland, School of Agriculture and Food Sciences, Brisbane, QLD 4072 Australia; 2 Department of Primary Industries, Yanco Agricultural Institute, Yanco, NSW 2703 Australia

**Keywords:** Rice, Aerobic production, QTL mapping, Root cone angle

## Abstract

**Background:**

Aerobic rice production (AP) may be a solution to the looming water crisis by utilising less water compared to traditional flooded culture. As such, development of genotypes with narrow root cone angle (RCA) is considered a key AP adaptation trait as it could lead to deeper rooting and ensure water uptake at depth. Quantitative trait loci (QTL) and genes associated with rooting angle have been identified in rice, but usually in conventional transplanted systems or in upland and drought conditions. This study aimed to identify QTL associated with RCA in AP systems using a recombinant inbred line population derived from IRAT109.

**Results:**

Four experiments conducted in glasshouse and aerobic field conditions revealed significant genotypic variation existed for RCA in the population. Single and multiple QTL models identified the presence of eight QTL distributed in chromosomes 1, 2, 3, 4, and 11. Combined, these QTL explained 36.7–51.2% of the genotypic variance in RCA present in the population. Two QTL, *qRCA1.1* and *qRCA1.3,* were novel and may be new targets for improvement of RCA. Genotypes with higher number of favourable QTL alleles tended to have narrower RCA. *qRCA4* was shown to be a major and stable QTL explaining up to 24.3% of the genotypic variation, and the presence of the target allele resulted in as much as 8.6° narrower RCA. Several genes related to abiotic stress stimulus response were found in the *qRCA4* region.

**Conclusion:**

Stable and novel genomic regions associated with RCA have been identified. Genotypes which had combinations of these QTL, resulted in a narrower RCA phenotype. Allele mining, gene cloning, and physiological dissection should aid in understanding the molecular function and mechanisms underlying RCA and these QTL. Ultimately, our work provides an opportunity for breeding programs to develop genotypes with narrow RCA and deep roots for improved adaptation in an AP system for sustainable rice production.

**Supplementary Information:**

The online version contains supplementary material available at 10.1186/s12284-021-00471-2.

## Background

Water availability for agricultural production is currently under threat, and is expected to decrease as a result of increasing population, higher food demand, and global climate change (Elliott et al. 2014; Mickelbart et al. 2015). The challenge now is to increase food and agricultural productivity while adapting to and mitigating the effects of climate change (Thorup-Kristensen et al. 2020). The interplay between water-intensive nature of traditional rice production grown under permanent water, looming water crisis, and increasing labour costs has shifted the attention to aerobic rice cultivation. As defined by Kato et al. (2009), aerobic rice production (AP) system is an intensive rice cultivation method which consists of direct seeded rice cultivation under non-flooded, well-watered conditions. The AP system was shown to use less than 50% of the irrigation water compared to conventional flooded rice in China (Bouman et al. 2007) in addition to reduced labour requirements (Huaqi et al. 2002) and greenhouse gas emissions in the Philippines (Alberto et al. 2009).

Root system architecture (RSA) is the arrangement of the crop root system in terms of specific geometric configuration across a rooting medium. RSA of crops is determined by several factors including branch distribution and magnitude, root growth angle, and root length (Abe and Morita 1994; Jung and McCouch 2013). RSA determines anchorage, soil nutrient and water exploitation, and developmental plasticity, and these qualities will have significant effect on maximum yield and yield stability. Root cone angle (RCA), a component of RSA, is the rooting angle of a plant relative to the vertical axis (Bettembourg et al. 2017) and is determined by measuring the two most external nodal roots of a plant, while root growth angle (RGA) is relative to the horizontal axis or soil surface, and as such, RCA and RGA are inversely related. Genotypes with narrow RCA (wider RGA) are expected to have deeper rooting systems, although variable results have been observed in terms of the relationship between RCA/RGA and rooting depth. By evaluating 12 cultivars in upland fields, Kato et al. (2006) suggested that RGA was associated with genotypic variation on development of deeper roots. Similarly, Uga et al. (2013a) also showed that genotypes with higher ratio of deep roots (RDR, increased RGA) tended to have increased rooting depth using Kinandang Patong (KP), IR64, and a near isogenic line derived from KP/IR64 cross with *DRO1*, in an upland field condition. On the contrary, Abe and Morita (1994) showed that this relationship was dependent on specific cultivars, in addition to environmental and agronomic factors. In an AP system, significant genetic correlations between percentage of deep roots and grain yield has been found, revealing the advantage of deeper roots in aerobic conditions (Mitchell et al. 2019). Plants in AP systems are typically exposed to transient water stress between irrigations in the upper soil profile, and as such, development of deeper roots allow the uptake of water at depth. In terms of phenotyping, rooting depth is relatively hard to determine while RCA is easier to measure with established phenotyping methods already in place (Courtois et al. 2013; Trachsel et al. 2011). The broad sense heritability of rooting angle traits has been shown to be moderate to high in rice and other cereals (Alahmad et al. 2019; Courtois et al. 2013). Additionally, large genetic variation also exist in rice germplasm for RCA (Bettembourg et al. 2017). It is therefore plausible to get good responses to selection for RCA. To be able to do this, a greater understanding of the genomic regions such as quantitative trait loci (QTL), genes, and pathways involved in the development of narrower RCA may help in breeding efforts to improve rice productivity especially for AP systems.

Several studies have been conducted in rice to investigate genomic regions associated with narrow root angle (Kitomi et al. 2015; Lou et al. 2015; Uga et al. 2015; Uga et al. 2013a; Uga et al. 2013b). Using RDR to shallow roots, as an index of RGA and rooting depth, *DRO1* was mapped in chromosome 9 and was subsequently cloned using KP, an upland cultivar, as the donor (Uga et al. 2013a). It was established that 1 bp-deletion in IR64 caused a premature stop codon in the gene and the transformation of KP containing candidate gene increased deep rooting. To date, *DRO1* is the only deep rooting gene cloned that was demonstrated to increase RDR thereby stabilising rice production in upland conditions and under drought stress. *DRO2*, *DRO3*, *DRO4*, and *DRO5* were also mapped from KP using different populations and genetic backgrounds. *DRO2* was mapped in chromosome 4 using three F_2_ populations of different genetic backgrounds- ARC5955 (*aus*), Pinulupot1 (*indica*), and Tupa729 (*tropical japonica*). *DRO3* is a QTL for RGA identified from KP and only affects the RGA of rice when *DRO1* functional allele is also present. *DRO4* and *DRO5* were identified and mapped in chromosome 2 and 6 respectively using three populations (Momiroman, Yumeaoba, and Tachisugata) and KP as the donor. It was established that RGA of rice genotypes with functional *DRO1* alleles were controlled by major QTL (*DRO2, DRO3,* and *DRO4*). Furthermore, another QTL for RDR, *qRDR-2*, was also identified from IRAT109 and was mapped on chromosome 2 (Lou et al. 2015). IRAT109, an upland tropical japonica cultivar originating from Côte d’Ivoire, Africa, has been shown to have robust root system (Qu et al. 2008). As a result, IRAT109 has been used extensively as a parent in QTL mapping experiments involving various root traits (Lou et al. 2015; Qu et al. 2008; Yue et al. 2006). The QTLs mentioned above were identified and characterised on paddy fields and usually with drought stress. To date, there has been limited studies involving the identification and characterisation of genomic regions associated with RCA for a high yielding aerobic cultivation system, where water availability is generally high but transient water deficit develops.

This study was conducted to identify stable and environment-specific genomic regions associated with rice RCA grown in AP system. A recombinant inbred line (RIL) population derived from IRAT109 was utilised and evaluated in glasshouse and aerobic field conditions. The detected QTL were compared with previously reported loci in literature to identify stable and novel genomic regions associated with RCA which may be exploited further for precise introgression in target cultivars for improved adaptation in AP systems.

## Methods

### Plant Materials

An elite Australian cold tolerant cultivar, Sherpa, was crossed with an upland tropical japonica cultivar, IRAT109. F_1_s between these cultivars were produced on two separate occasions. Resulting F_1_s were then self-pollinated to produce F_2_s, and subsequently, single seed descent method was carried out until F_6_ generation to produce the RILs. A total of 252 RILs derived from Sherpa/IRAT109 were genotyped and evaluated for RCA at the University of Queensland (UQ) Gatton (27.5551° S, 152.3369° E) and St Lucia (27.4975° S, 153.0137° E) campus.

### SNP Genotyping

The RILs (252) were genotyped using the Diversity Arrays Technology (DArT) genotyping-by-sequencing platform (DArTSeq). Initially, there were a total of 254 RILs, but subsequent analyses revealed two pairs of genotypes were identical based on single nucleotide polymorphism (SNP) data. In the succeeding analyses carried out (both genotype and phenotype data), these identical pairs were treated as one, making the final number of RILs to 252. DArTSeq allowed for complexity reduction and sequencing of low copy sequences (corresponding predominantly to active genes) through the use of methylation sensitive restriction enzymes. These libraries were then sequenced using a next generation sequencing platform (Illumina HiSeq 2500) and the resulting sequences were aligned to *Oryza sativa* v7.0 reference genome (https://jgi.doe.gov/) to identify SNPs. The R package dartR (Gruber et al. 2018) was used to filter SNPs called from the DArTSeq data. Missing SNP data were imputed using softImpute (Hastie et al. 2015) R package. In summary, polymorphic SNPs between Sherpa and IRAT109 were identified, and SNP loci with call rates below 80% and a minor allele frequency (MAF) of < 5% were omitted from the genotype data. Additionally, markers deviating from the expected allele frequencies of RILs were also dropped.

### RCA Phenotyping

A total of four experiments were conducted to evaluate RCA. One experiment was conducted in a glasshouse at UQ St Lucia with controlled temperatures while the other three experiments were carried out in the field at UQ Gatton. The experiments were part of a wider evaluation program, and as such carried a large number of non-RIL genotypes for evaluation. The parents and Australian standard, Reiziq, were present in all experiments. The number of RIL genotypes varied from 234 to 250 depending on seed availability.

The glasshouse experiment (GH) was conducted following the clear pot method inspired by Richard et al. (2015) for RCA determination, with some modifications. In brief, single seeds of RILs and check varieties were direct seeded in 4 L clear pots (ANOVApot®, 200 mm diameter, 190 mm height, http://www.anovapot.com/php/anovapot.php) filled with pine bark potting media (70% composted pine bark 0–5 mm, 30% coco peat, pH 6.35, EC = 650 ppm, nitrate = 0, ammonia < 6 ppm and phosphorus = 50 ppm) with 3 g/L Osmocote Exact 3-4 M (19–9-10 + 2MgO + TE, ICL Specialty Fertilizers), 2 g/L Osmocote Exact 5-6 M (15–9-12 + 2MgO + TE) and 0.82 g/L Suscon Maxi Green (Nufarm, Australia). Seeds were sown vertically at a depth of 3 cm along the pot wall with a final density of 12 plants per pot. To facilitate root growth along the wall of the pot, the seeds were seeded with lemma (embryo) oriented downwards facing the wall. The clear pots were then inserted inside a similar sized 4 L black pots to prevent light penetration. Pots were watered overhead with deionised water every 2–3 days. The experiment was carried out in a temperature-controlled glasshouse set at 28 °C/21 °C day/night temperatures with natural light. RCA was measured manually 35 days after sowing (DAS) using a protractor, by measuring the cone angle from the two most external nodal roots. In total, 282 genotype entries, including 245 RILs, were evaluated. The parent genotypes as well as an Australian standard had 6 replications while the remaining genotypes were replicated twice and arranged in a randomised complete block design.

The three field experiments were conducted in two seasons, 2018–2019 and 2019–2020. An intermittent water stress (IWS) field experiment (IWS19) was conducted in the 2018–2019 season with a partially-replicated design (278 genotypes, 250 RILs, 43% replication) while both well-watered (WW) and IWS experiments were conducted in 2019–2020 season (WW20 and IWS20) with resolvable column design with two replications (246 genotypes, 234 RILs) in each experiment. The designs were generated using R package DiGGer (Coombes 2009). IWS19 and WW20 were irrigated three times a week (Monday, Wednesday and Friday) while IWS20 was irrigated twice a week (Tuesday and Friday), with 24 mm per irrigation event. In IWS19 a solid set sprinkler irrigation was utilised, while in WW20 and IWS20 irrigation was applied via a lateral boom irrigation. Due to differences in application methods the IWS19 was considered sub-optimal, compared to the lateral boom irrigation in WW20. IWS19, IWS20, and WW20 received a total of 9.6, 10.7, and 16.6 megaliters/ha of combined irrigation and rainfall, respectively.

IWS19 consisted of plots of 1 m in length with six rows at an inter-row spacing of 0.2 m, and planted at 180–220 seeds m^− 2^ with a single row push seeder. Basal fertiliser was applied 5 days prior to sowing at a rate of 480 kg/ha using Incitec Pivot’s Pastureboosta (24–4–13-4) fertiliser with an additional 15 kg/ha of zinc sulphate monohydrate and was lightly incorporated. An additional 80 kg/ha of nitrogen was applied at 52 DAS in the form of urea. Pre-emergent herbicide treatment was carried out 5 days after initial irrigation. The treatment consisted of recommended rates: (1) Stomp 440 (440 g/L Pendimethalin, CropCare), (2) Magister (480 g/L Clomazone; FMC Australia), (3) Gramoxone (250 g/L Paraquat, Syngenta).

In WW20 and IWS20, seeds were drill sown to a depth of 3–4 cm at a rate of 130 kg/ha which is considered as industry recommendation. Plots were 2 m in length with seven rows and an inter-row spacing of 0.22 m, resulting in a 3.08 m^2^ plot size. Basal fertiliser was applied as 400 kg/ha of Incitec Pivot’s CK140S (23–2–18-4) and 15 kg/ha of zinc sulphate monohydrate, and nitrogen topdressing occurred at 42 and 54 DAS for the WW and IWS, respectively. Furthermore, IWS20 had addition foliar application of iron sulphate (20 g/L iron sulphate+ 6 g/L urea at 150 L/ha) at 89 DAS. Pre-emergent herbicide regime was the same as IWS19, however Sempra® (750 g/kg Halosulfuron-Methyl, Nufarm) was also applied at 56 DAS in IWS20.

**Fig. 1:**
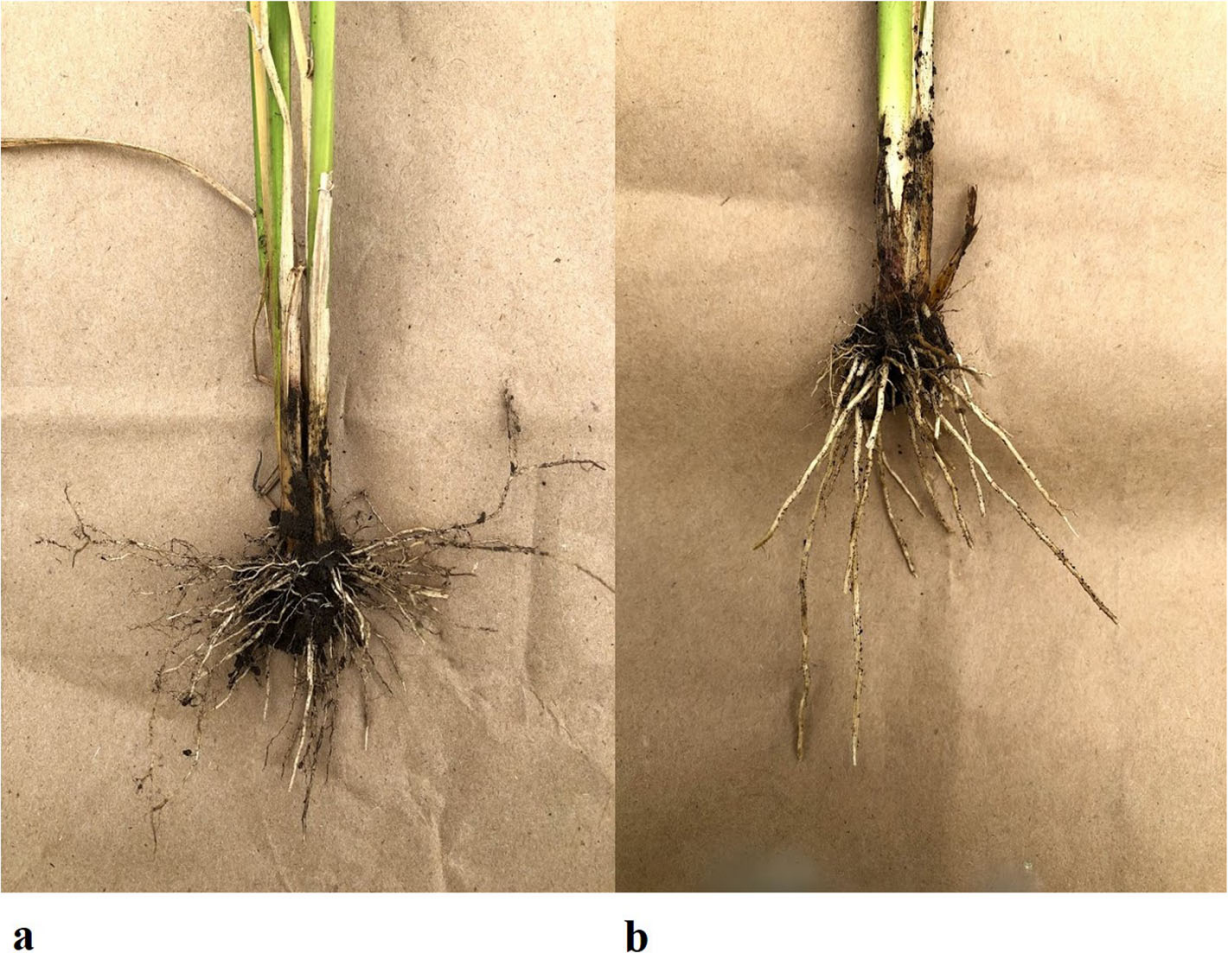
Root cone angles of a (**a**) wide-angled genotype and (**b**) narrow-angled RILs from WW20 experiment

Root crowns were manually removed out of the plots, ~ 99 DAS, after majority of the genotypes had reached the heading stage (IWS19 and WW20) and after maturity (IWS20). Two representative plants were selected from an internal row. The plants were lifted from the ground along with soil attached. Soil particles were manually removed from the roots to facilitate the measurement of RCA (Fig. 1) similar to the procedure carried out in GH.

### Statistical Analysis

A multiplicative mixed linear model was used for the analysis which was implemented in ASReml (V4.1; VSNi, UK) package running in the R environment (R Core Team 2019). The parents, Reiziq and RIL entries that were present in at least two environments were treated as a random effect, while all other entries were treated as a fixed effect. Best linear unbiased predictors (BLUPs) were obtained after accounting for randomisation process and spatial variation. A multi-environmental (MET) analysis was undertaken where genotype by environment was modelled using an unstructured variance-covariance matrix. BLUPs for each genotype across trial combination were predicted. Generalised heritability for individual experiments were calculated according to Smith et al. (2006). Heritability was calculated twice using the variance parameters estimated from the single experiment and MET analysis to describe the improvement in the predictions.

### Linkage Map Construction and QTL Mapping

Linkage map was constructed from the final filtered genotype data using ASMap (Taylor and Butler 2017) and R/qtl package (Broman et al. 2003). Markers with (1) identical genotype data, (2) potential evidence for genotyping errors (Lincoln and Lander 1992), (3) located on the same position, and (4) with missing data greater than 5% were dropped from the genotype data. Genetic distances between markers, in centiMorgans (cM) were estimated using the Kosambi map function of ASMap package (Taylor and Butler 2017). The final linkage map was estimated using the Lander-Green algorithm (Lander and Green 1987). Conditional genotypic probabilities were computed using 1 cM step distance and then genotypes were simulated given observed marker data by utilising sim.geno function with 32 replications and then single and multiple QTL model analysis were carried out using the Haley-Knott regression method (Haley and Knott 1992) using BLUPs computed from the MET model. For both QTL models, the logarithm of odds (LOD) threshold used to report QTL were based on the results of 1000 permutations at a 5% significance level. Bayesian credible confidence intervals around each significant QTL peak were determined using bayesint function of R/qtl. Two-dimensional scans with two-QTL and multi-QTL model, similar with composite interval mapping, to identify multiple and interacting QTL were carried out using stepwiseqtl, refineqtl, and fitqtl functions in R/qtl. The estimated effects and R^2^ by each and all QTL were also estimated. Linear regression was carried out using R to test for the significance of pyramiding identified QTL in respective experiments.

## Results

### RCA Genotypic Variation

**Fig. 2:**
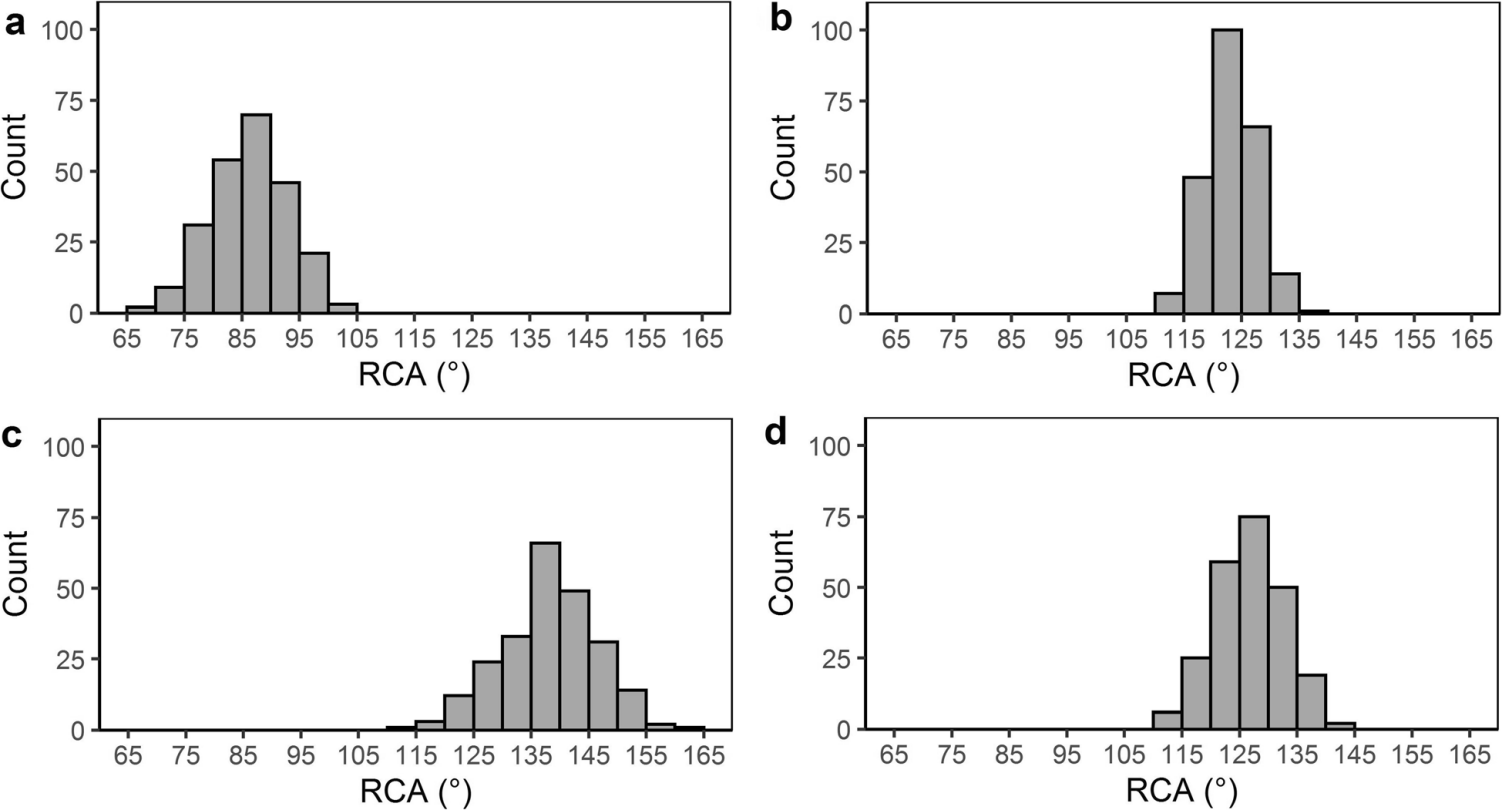
Distribution of the RCA obtained from Sherpa/IRAT109 recombinant inbred lines. **a** GH - Glasshouse Experiment; **b** IWS19 - intermittent water stress 2019 experiment, **c** WW20 - well-watered 2020 experiment, and **d** IWS20 - intermittent water stress 2020 experiment. RCA- root cone angle

Of the 252 RILs, 245, 250, 234, and 234 were phenotyped for RCA in GH, IWS19, WW20, and IWS20, respectively. Of these, a total of 236 (93.7%) RILs were present in at least two environments and were retained in the MET analysis. Highly significant (*P* < 0.001) genotypic variation in RCA was found in RILs across three experiments conducted (GH, WW20, and IWS20), and significant (*P* < 0.05) variation was found in IWS19. RILs in GH experiment had narrower RCA than in the field and ranged from 68°-104° (Fig. 2a), with a mean of 86°. In the IWS19 field experiment, RCA ranged from 111°-137°, with a mean of 123° (Fig. 2b). In WW20 and IWS20, a greater range in RCA existed among RILs, compared to IWS19, with wider RCA observed (Table 1) in WW20 (Fig. 2c) and IWS20 (Fig. 2d). Single trial heritability ranged from 0.14–0.48, while an improved heritability was observed in MET analysis (Table 1).

**Table 1:** RCA (°) statistics for RILs derived from Sherpa/IRAT109 evaluated in four experiments

	GH	IWS19	WW20	IWS20
	*n* = 245	*n* = 250	*n* = 234	n = 234
	RILs			
Min	68	111	114	110
Max	104	137	161	145
Mean	86**	123*	138**	127**
Single trial heritability	0.38	0.14	0.48	0.42
MET heritability	0.47	0.42	0.62	0.62
	Parents			
IRAT109	80	117	127	118
Sherpa	88	125	141	129
	RCA Groups			
Mean top 10% narrowest RCA	75	115	123	116
Mean top 10% widest RCA	97	131	152	137

*RCA* root cone angle, *GH* Glasshouse Experiment, *IWS19* intermittent water stress 2019 experiment, *WW20* well-watered 2020 experiment, and *IWS20* intermittent water stress 2020 experiment; ** - *P* < 0.001; *- *P* < 0.05; MET- multi-environment trial

In terms of the parents, IRAT109 consistently showed narrower RCA compared to the recipient parent, Sherpa (Table 1). Among the field experiments, WW20 had the highest difference between the parents, with IRAT109 having an RCA of 127° while Sherpa had 141°. Transgressive segregation was also observed in all the experiments conducted. Consistent among the experiments, the top 10% narrowest RILs had narrower RCA compared with IRAT109, and the top 10% widest RCA RILs produced wider root angle compared with Sherpa.

Strong positive genetic correlations existed for RCA between the experiments. The strongest correlation with *r* = 0.95 existed between IWS19 and IWS20, while between water regimes in WW20 and IWS20 (*r* = 0.86), and between IWS19 and WW20 (*r* = 0.72). There was also a positive relationship observed between the glasshouse and field experiments conducted. GH showed a moderate positive correlation of *r* = 0.61, *r* = 0.56, and *r* = 0.44 with WW20, IWS20, and IWS19 respectively.

### SNP Genotyping and Linkage Map Construction

DArTSeq resulted in a total of 6508 SNP loci distributed across the rice genome. After filtering for loci with low call rates, MAF, and polymorphic between Sherpa and IRAT109, a total of 2624 high quality SNP loci were retained in the dataset (Fig. S1). Analysis of the markers revealed the number of SNP markers ranged from 106 (chromosome 9) to 446 (chromosome 1) per chromosome, with an average of around 219 markers. In terms of physical distance, the average distance in between markers ranged from 93.74 kb (chromosome 1) to 230.93 kb (chromosome 2), with an average distance of 153.06 kb across the rice genome (Table S1). Identification of redundant markers was carried out to find markers that were completely correlated. After the execution of filtering algorithms described above, a total of 1394 markers were retained in the final dataset. Chromosome 1 still had the highest number of markers with 268, while chromosome 9 had the lowest with a total of 49 markers. Distance wise, chromosome 1 also had the average distance of closest markers, with a mean of 156.14 kb apart, while chromosome 9 had the largest average distance of 459.35 kb.

The final SNP data consisting of 1394 markers was utilised to construct the linkage map. These markers covered a total of 1460.5 cM of rice genome using the Kosambi map function (Fig. S2). Similar to the physical map length, chromosome 1 was the longest with 179.6 cM while chromosome 10 was the shortest with a total length of 79.1 cM. The largest inter-marker distance was 36.0 cM, observed in chromosome 2. The mean distance in between markers across the whole genome was 1.1 cM. The final linkage map constructed and utilised in this study was in congruence with both the length of chromosomes in terms of physical distance and with previously constructed linkage maps (Harushima et al. 1998). Additionally, using this final SNP data, the RILs were genotyped with an average of 97.4% of the markers across the genome. Also, on average, RILs have 51.9% Sherpa (AA) allele, and 48.1% IRAT109 (BB) allele.

### Genomic Regions Associated with RCA

**Fig. 3:**
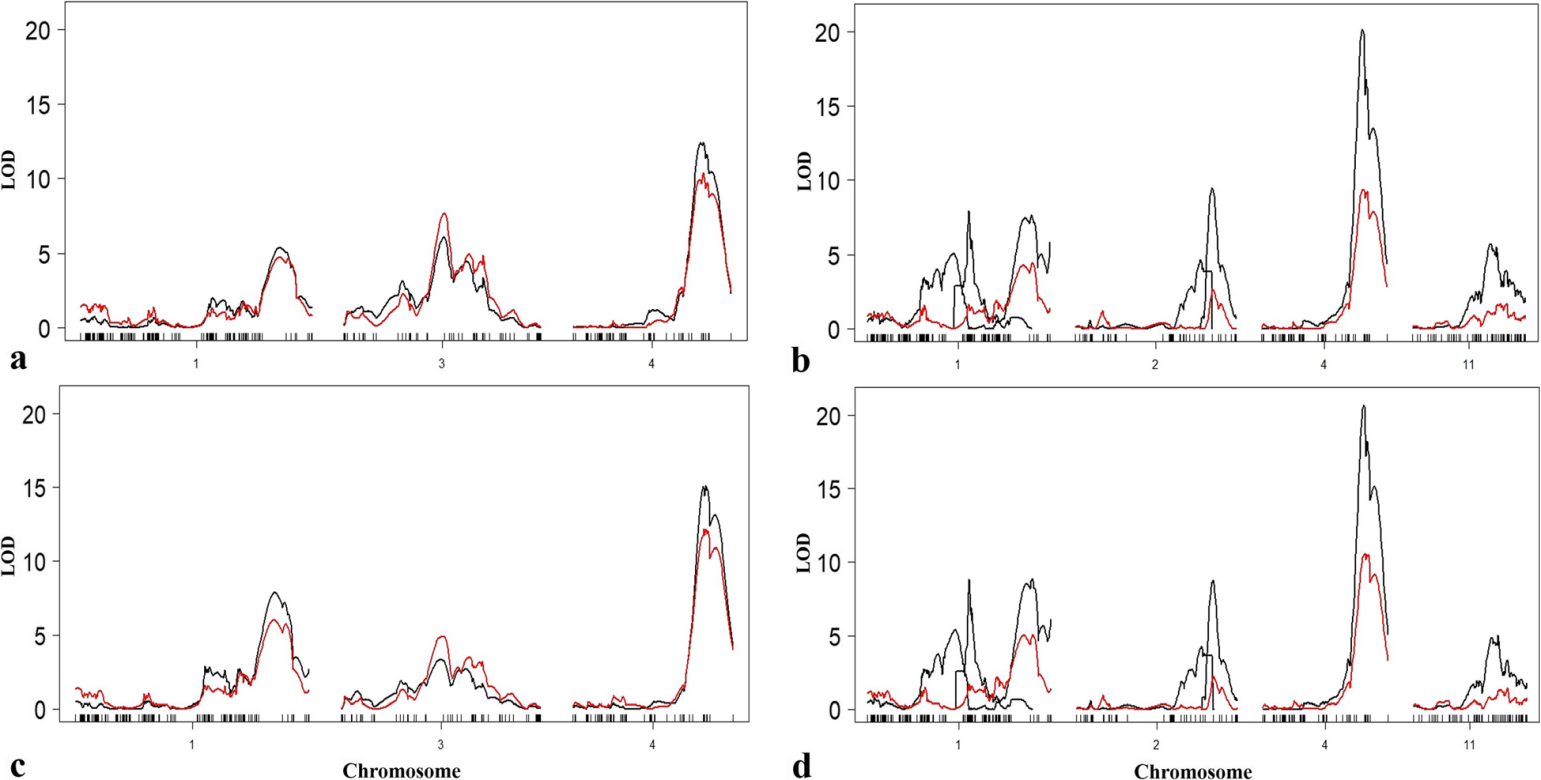
LOD profiles for QTL associated with RCA detected using single and multiple QTL models. Red line – single QTL LOD profile; black line – multiple QTL LOD profile; **a** GH - Glasshouse Experiment; **b** IWS19 - intermittent water stress 2019 experiment, **c** WW20 - well-watered 2020 experiment, and **d** IWS20 - intermittent water stress 2020 experiment; RCA- root cone angle; LOD- logarithm of odds; QTL- quantitative trait loci

Using the constructed linkage map and phenotype data, two dimensional scans and analysis for multiple-QTL models identified significant genomic regions located on chromosomes 1 and 4 associated with RCA, similar to the results compared with single QTL model (Table 2, Fig. 3). With the final QTL models in each environment, no significant interactions between genomic regions were identified. Similar with the single QTL model, the genomic region on chromosome 4 (*qRCA4*) was consistent and registered the highest LOD scores in all the experiments (Table 2). This QTL was located around 97.0–104.6 cM (29.78–30.69 Mb, LOD: 12.45–20.70) and was able to explain about 17.40–24.28% of the genotypic variation present for RCA in the population. Another novel QTL detected across all the experiments was located in chromosome 1 (*qRCA1.1*, pos: 148.7–164.9 cM, LOD: 5.37–9.35). This was a minor QTL which explained 6.99–10.22% of the variation in RCA in the population. Introgression of the IRAT109 allele of the QTL from chromosome 4 resulted in a narrower RCA, ranging from 4.94–8.58°, while IRAT109 allele from the chromosome 1 QTL resulted in a wider RCA, ranging from 2.78–7.36°.

**Table 2:** Putative QTL associated with RCA identified using Sherpa/IRAT109 RIL population

Experiment	QTL	Chr	Nearest Marker	Mb	cM	CI (cM)	LOD	AE	Favourable Allele	R^2^
GH	*qRCA1.1*	1	Chr1_39524707	39.52	154.60	148.7–162.4	5.37	2.10	Sherpa	6.99
	*qRCA3*	3	Chr3_16551946	16.55	78.23	71.6–81.0	6.10	−1.90	IRAT109	8.00
	*qRCA4*	4	Chr4_30036160	30.03	101.43	97.0–104.0	12.45	−2.97	IRAT109	17.40
										(36.73)
IWS19	*qRCA1.2*	1	Chr1_19340596	19.34	84.87	67.2–89.8	5.06	−1.73	IRAT109	5.22
	*qRCA1.3*	1	Chr1_24919134	24.92	99.88	99.0–100.5	7.94	1.81	Sherpa	8.42
	*qRCA1.1*	1	Chr1_39416289	39.42	161.41	150.7–164.9	7.66	1.39	Sherpa	8.10
	*qRCA2.1*	2	Chr2_27972049	27.97	122.53	119.0–133.2	4.64	1.36	Sherpa	4.76
	*qRCA2.2*	2	Chr2_30400561	30.40	134.12	131.4–135.9	9.48	−1.92	IRAT109	10.22
	*qRCA4*	4	Chr4_29775386	29.78	98.97	97.0–99.9	20.13	−2.47	IRAT109	24.18
	*qRCA11*	11	Chr11_19591983	19.59	76.31	73.4–83.3	5.74	−1.16	IRAT109	5.96
										(49.72)
WW20	*qRCA1.1*	1	Chr1_39524707	39.52	153.61	148.7–162.4	9.35	3.68	Sherpa	10.22
	*qRCA3*	3	Chr3_16551946	16.55	76.34	44.6–100.3	3.31	−1.86	IRAT109	4.12
	*qRCA4*	4	Chr4_30366129	30.37	102.14	99.0–104.6	15.51	−4.29	IRAT109	21.11
										(38.31)
IWS20	*qRCA1.2*	1	Chr1_23120475	23.12	85.86	78.0–90.8	5.40	−2.44	IRAT109	5.42
	*qRCA1.3*	1	Chr1_24919134	24.92	99.88	99.0–100.5	8.84	2.63	Sherpa	9.18
	*qRCA1.1*	1	Chr1_39416289	39.42	161.41	152.6–164.9	8.90	1.97	Sherpa	9.25
	*qRCA2.1*	2	Chr2_27972049	27.97	122.53	119.0–133.2	4.30	1.71	Sherpa	4.27
	*qRCA2.2*	2	Chr2_30400561	30.40	134.12	131.4–135.9	8.79	−2.42	IRAT109	9.12
	*qRCA4*	4	Chr4_29775386	29.78	98.97	97.0–99.9	20.70	−3.28	IRAT109	24.28
	*qRCA11*	11	Chr11_20763446	20.76	82.62	73.4–84.0	5.02	−1.38	IRAT109	5.03
										(51.22)

Mb - physical map position of each marker based on the Nipponbare sequence at RAP database (http://rapdb.dna.affrc.go.jp/); CI- bayesian credible confidence intervals in centiMorgan (cM); AE: additive effect of the allele from IRAT109 compared with that from the recipient line, Sherpa; R^2^ - percentage of the genotypic variance explained by each QTL. Numbers in parentheses indicate percentage of the variance explained by multiple QTL; GH - Glasshouse Experiment; IWS19 - intermittent water stress 2019 experiment, WW20 - well-watered 2020 experiment, and IWS20 - intermittent water stress 2020 experiment

Aside from these stable QTL mentioned above, six environment-specific genomic regions associated with RCA were also identified in chromosomes 1, 2, 3, and 11 (Table 2) using the multiple QTL model approach. It was noted that compared with the results from the single QTL model, genomic regions located on chromosomes 3 and 12 (Fig. S3) previously significant for IWS19 and IWS20 became insignificant and were therefore dropped from the final multiple QTL model. Moreover, compared with the single QTL model, the multiple QTL model identified additional genomic regions associated with RCA. Five genomic regions located on chromosomes 1, 2, and 11 (*qRCA1.2, qRCA1.3, qRCA2.1, qRCA2.2,* and *qRCA11*) were found to be significant in the intermittent water stress environments, IWS19 and IWS20. Detecting similar genomic regions acting on these two experiments seems sensible since these were both regarded as IWS environments and there was also strong positive genetic correlations between the two. Looking closer into the QTL, the pairs of QTL identified on chromosomes 1 and 2 were located in close proximity with each other, 14.0 and 11.6 cM apart, respectively. In both instances, introgression of IRAT109 alleles from these regions resulted in contrasting effects with RCA, thereby nullifying the effect when being considered in the single QTL model. Finally, *qRCA11,* on the long arm, was also found to be associated with RCA in the abovementioned experiments and the donor allele resulted in a narrower RCA of about 2.32°-2.76°. Looking into the more favourable water availability GH and WW20 experiments, a genomic region specific to these experiments was also identified in chromosome 3 (*qRCA3*). This region was similar to that identified from the single QTL model, and its introgression resulted in about 3.72°-3.80° narrower RCA.

Combining the QTL analyses for RCA from the four experiments, a total of eight genomic regions associated with RCA in aerobic conditions were found on chromosomes 1, 2, 3, 4, and 11. Using different models, consistent genomic regions were identified in chromosomes 1 and 4 across all the experiments conducted. IRAT109 allele from the *qRCA1.1* increased RCA while it decreased RCA in *qRCA4*. Additionally, environment specific QTL were also detected on chromosomes 1, 2, 3, and 11. More importantly, *qRCA4* appeared to be consistently associated with narrower RCA, regardless of whether the analysis was carried out in single or in multiple QTL models and hence has been identified as a valuable genomic region for aerobic adaptation.

### Benefit of Stacking QTL Identified

Using the genotype information of RILs in the peak markers of identified QTL, the allelic composition and the number of genomic regions with favourable alleles for each RIL was determined. A simple regression analysis between the number of favourable alleles present and the BLUPs revealed a significant benefit of stacking QTL (*r*^*2*^ = 0.30–0.32**, *P* < 0.001). In the relatively well-watered environments, the narrowest RCA was achieved when *qRCA1.1, qRCA3.1* and *qRCA4.1* were combined (Table 3). The combination of these three QTL resulted in 7.8–11.0° narrower RCA compared to the mean RCA of genotypes with single QTL. In the case of the seven QTL in the IWS environments, there was the potential for 128 QTL combinations all of which were not present in the RIL population. Thus, inspection of the relative effects of the QTL in IWS environments as well as all the combinations available in the RILs was undertaken which revealed four QTL (*qRCA1.1, qRCA2.1, qRCA2.2,* and *qRCA4*) were the most valuable from the identified seven QTL (Table 4). The presence of these four QTL together also resulted in narrower RCA (8.2–11.5°) compared to the genotypes having single QTL.

**Table 3:** RCA (°) of RILs with different QTL combinations in GH and WW20 environments

QTL Combination	N	GH		WW20	
		RCA	SE	RCA	SE
No QTL	24	93.8	1.0	147.0	1.3
*qRCA1.1*	17	89.8	1.1	142.8	1.6
*qRCA3*	18	88.5	1.5	143.2	1.2
*qRCA4*	48	87.9	0.7	139.7	0.8
*qRCA1.1 + qRCA3*	20	87.1	1.3	140.0	1.5
*qRCA1.1 + qRCA4*	21	85.1	1.2	134.0	1.5
*qRCA3 + qRCA4*	43	84.1	0.9	135.9	1.1
*qRCA1.1 + qRCA3 + qRCA4*	45	80.9	0.8	130.9	1.1

*RCA* root cone angle, *RIL* recombinant inbred line, *QTL* quantitative trait loci, *GH* glasshouse experiment, *WW20* well-watered 2020 experiment, *N* number of genotypes, *SE* standard error of the mean

**Table 4:** RCA (°) of RILs with different QTL combinations in IWS19 and IWS20 environments

QTL Combination	N	IWS19		IWS20	
		RCA	SE	RCA	SE
No QTL	8	129.5	1.4	135.1	1.7
*qRCA1.1*	3	126.6	1.8	131.4	2.8
*qRCA2.1*	15	126.9	0.9	131.9	1.2
*qRCA2.2*	13	125.6	1.3	130.6	1.6
*qRCA4*	13	124.6	0.8	128.7	1.1
*qRCA1.1 + qRCA2.1*	20	124.6	0.9	128.8	1.1
*qRCA1.1 + qRCA2.2*	13	125.3	0.9	129.8	1.2
*qRCA2.1 + qRCA2.2*	6	124.9	1.4	129.5	1.6
*qRCA1.1 + qRCA4*	3	124.7	1.5	127.7	2.2
*qRCA2.1 + qRCA4*	40	123.9	0.6	127.6	0.7
*qRCA2.2 + qRCA4*	30	122.3	0.8	125.7	1.0
*qRCA1.1 + qRCA2.1 + qRCA2.2*	1	113.4	(−)	114.7	(−)
*qRCA1.1 + qRCA2.1 + qRCA4*	30	121.0	0.6	123.4	0.9
*qRCA1.1 + qRCA2.2 + qRCA4*	25	119.7	0.8	122.2	1.1
*qRCA2.1 + qRCA2.2 + qRCA4*	8	119.9	0.9	122.6	1.2
*qRCA1.1 + qRCA2.1 + qRCA2.2 + qRCA4*	8	117.5	1.5	119.0	2.0

*RCA* root cone angle, *RIL* recombinant inbred line, *QTL* quantitative trait loci, *IWS19* intermittent water stress 2019 experiment, *IWS20* intermittent water stress 2020 experiment, *N* number of genotypes, *SE* standard error of the mean

## Discussion

This paper evaluated a RIL population derived from a genotype with known narrow RCA, IRAT109, in both glasshouse and field conditions spanning 2 years. The RCA values obtained from the glasshouse experiment ranged from 78°-104°, while the field experiments ranged from 114°-161° in WW environments and 110°-145° in IWS environments. This range in RCA was narrower but within the range obtained by Bettembourg et al. (2017) who evaluated a diverse rice germplasm set consisting of *indica* and *japonica* cultivars. The difference in the range obtained may be due to the nature of genetic materials utilised in this study. Using a set of 166 *japonica* genotypes, a range of 36°-164° RCA using the Rhizoscope phenotyping system was reported (Bettembourg et al. 2017). In this present study, genetic correlation analysis conducted between the experiments also showed moderate to strong positive relationships between RCA measured across experiments, including between glasshouse and field experiments. To our knowledge, this is the first report showing the utility of a clear pot method to evaluate rice RCA by measuring RCA of nodal roots in early growth stages. The utility of a similar method was first shown in wheat and barley by measuring seminal root angle (Richard et al. 2015; Robinson et al. 2016). Positive genetic correlations between the glasshouse and field experiments was indicative that screening for RCA of nodal roots in early growth stages can be used as a selection method for genotypes to be tested in the field, similar to what has been observed in the seminal root angle screening and RSA in wheat (Manschadi et al. 2008). The phenotyping system evaluating RCA based on nodal roots, around 35 DAS has been established here as a high throughput method for providing valuable phenotypic and genotypic RCA discrimination pertinent to AP production environments.

Linkage mapping utilising RILs derived from parents with contrasting root angle detected genomic regions associated with RCA in both glasshouse and field experiments. In terms of RCA, Bettembourg et al. (2017) suggested that a linkage mapping approach may be a better tool to identify genomic regions associated with the trait rather than genome wide association (GWAS) mapping. In their study, subpopulations in a *japonica* panel they utilised showed only one-tail extremes and a very low number of extreme accessions, which will ultimately result in the SNPs being filtered out and by extension, undetectable QTL (Verdeprado et al. 2018; Zhang et al. 2016). In our study, using single and multiple QTL linkage mapping models, three QTL each have been identified in GH and WW20 experiments, while seven QTL were identified each in IWS20 and IWS19. Of the total eight QTL identified across all environments, six (*qRCA1.2, qRCA2.1, qRCA2.2, qRCA3, qRCA4*, and *qRCA11*) have been previously reported to be associated with root angle (Bettembourg et al. 2017; Lou et al. 2015; Uga et al. 2013b) while the other two (*qRCA1.1* and *qRCA1.3*) were novel and were first reported in this study (Fig. S4). *qRCA1.2* and *qRCA3* were mapped in relatively large genetic distance and as such, they may be less reliable. Given that their effects were also minor, these loci were considered of low priority. Nevertheless, genotyping with additional markers, such as simple sequence repeats and insertion/deletion markers, to saturate the region may provide further clarity in these loci. Of the eight QTL identified, *qRCA1.1* (39.42–40.41 Mb) and *qRCA4* (29.78–30.69 Mb) were shown to be stable across environments. The donor allele for narrow RCA in *qRCA1.1* was from Sherpa, while the *qRCA4* locus was from IRAT109. It is highly plausible that the allele frequency of this beneficial allele of *qRCA1.1* is already high in current Australian germplasm and is therefore more accessible to the breeding programs. On the other hand, *qRCA4* was shown to contribute narrower RCA and had the largest contribution to the total genotypic variance. To our knowledge, for the first time, this study also reports a major, stable QTL associated with rice RCA grown in AP systems in both glasshouse and field conditions. Several experiments conducted in transplanted systems or in artificial growth media have implicated these regions to be associated with rooting angle and other root-related traits. Using F_2_ populations derived from three cultivars and KP as the donor genotype, the same region on chromosome 4 (28.76–30.69 Mb), *DRO2*, was shown to be associated with RDR (Uga et al. 2013b), with RM6089 as the nearest marker. Using GWAS approach, Bettembourg et al. (2017) also identified a QTL on chromosome 4 located at 30.76 Mb from a japonica panel genotyped using a high density array. It is worth noting however, that this QTL was absent when the analysis was extended in an *indica* panel suggesting the absence or possibly presence at low frequencies of this particular QTL in the *indica* subgroup. The results of the present study provides us with confidence that *qRCA4* would have the potential utility for both *indica* and *japonica* breeding programs. Although *qRCA4* has been previously implicated with rooting angle in hydroponics and controlled environments (Bettembourg et al. 2017; Uga et al. 2013b), its identification in field conditions in this study further cemented its value for the improvement of narrow RCA in multiple AP environments. A closer inspection of *qRCA4* locus established that there were a total of 126 genes in the region using the MSU Osa1 Rice Gene Models database (Ouyang et al. 2007). Seven of these genes were enriched genes related to abiotic stimulus (LOC_Os04g49930, LOC_Os04g49970, LOC_Os04g49980, LOC_Os04g50820, LOC_Os04g50880, LOC_Os04g50990, and LOC_Os04g51330). There were also some notable genes encoding for transcription factors which are associated with endogenous stimuli. These information and potential candidate genes can aid in future molecular marker development activities and ultimately, identification of gene/s responsible to the target trait: narrow RCA. Additionally, using the 3 K Rice Genome Project (3KRGP) 400 K SNP database (Mansueto et al. 2017), a total of 644 SNPs were identified within the region. Using this data, priority SNPs can be identified such as those residing in (1) coding regions causing synonymous/non-synonymous mutation, (2) 3′ and 5′ UTR, (3) exonic region and (4) promoter region. Since IRAT109 was included in the 3KRGP, its allele can be compared to other target or known genotypes and marker development can be tailored based on this information along with allele frequencies.

Of particular interest was that under IWS experiments, QTL with relatively higher additive effects compared to other regions were identified. This suggests that along with stable QTL identified above (*qRCA1.1* and *qRCA4*), these IWS environment-specific QTL are also excellent targets for improvement of RCA for AP. It was also shown that genotypes possessing higher number of favourable QTL alleles tended to have narrower RCA. It has to be noted though that the analysis for stacking favourable QTL alleles was carried out in the RILs with non-uniform genetic background and may have led to over or under estimation of QTL effects, especially minor QTL. Development of near isogenic lines prior to QTL pyramiding will aid in the precise determination of QTL effects, in single or pyramided state, similar to what has been shown in brown planthopper resistance genes (Jena et al. 2017). Taken together, by implementing multiple QTL mapping strategies (single and multiple QTL models in R/qtl), we have identified a major and stable QTL across different environments/experiments and have also identified with high confidence novel QTL with moderate effects. The identification of these stable and novel genomic regions may provide value to breeding programs around the globe which target AP systems.

Currently, the RILs derived from Sherpa/IRAT109 cross are being tested for yield in AP systems. In terms of AP systems, it remains to be shown if similar effects achieved with *DRO1*, which increased yield under drought and upland conditions, can be revealed by introgressing *qRCA4* into different genetic backgrounds. To facilitate its inclusion in routine marker assisted selection programs, it is imperative that its effect and the robustness of the marker is demonstrated in multiple genetic backgrounds (Cobb et al. 2019). Segregating populations in three different genetic backgrounds were developed and are currently being evaluated to validate the effect of *qRCA4*. If successful, this could lay the foundations to develop high throughput molecular markers targeting this region. To date, there is very limited information available with regards to molecular markers related with QTL associated with root angle and depth possibly due to limited databases and sequence information available when these QTL were identified previously. With the advent of databases such as 3KRGP and other comparative genomics tools, development of molecular markers is now more attainable and should pave the way for the potential introgression of this locus into breeding programs when pertinent to the target production environment.

## Conclusions

We have identified eight QTL associated with rice RCA in an AP system, two of which were novel and have not been previously reported. When these favourable QTL were in combination, there was additional advantage by producing genotypes with narrower RCA. *qRCA4* is a promising major QTL that has been shown to be associated with narrow RCA which was consistently detected across experiments, and thus, it has a high potential in improving RSA of rice in the context of aerobic production. Detailed physiological dissection of RCA and the relationship of this QTL with grain yield and other physiologically important traits related to photosynthetic rates and metabolic activities under mild water stress conditions will shed some light on mechanisms related to its action. Finally, allele mining, haplotype analysis using publicly available databases, and gene cloning will enable breeding for improved rooting angle and indirectly depth, through marker-assisted selection and the elucidation of its molecular function.

## Supplementary Information

**Additional file 1: Table S1**. Summary of the number of detected polymorphic SNPs between Sherpa and IRAT109 and their average distances per chromosome. Numbers in parentheses are number of SNPs and average distance in cM using the final 1394 set of markers.

**Additional file 2: Fig. S1.** Graphical genotype of the 2624 polymorphic markers detected between Sherpa and IRAT109 using DaRTSeq.

**Additional file 3: Fig. S2.** Linkage map constructed from 1394 markers using RILs derived from Sherpa and IRAT109. (JPEG 101 kb)

**Additional file 4: Fig. S3.** LOD profiles for QTLs associated with root cone angle detected using single QTL model for (a) GH, (b) IWS19, (c) WW20, and (d) IWS20. Dotted blue line indicates LOD threshold. GH - Glasshouse Experiment; IWS19 - intermittent water stress 2019 experiment, WW20 - well-watered 2020 experiment, and IWS20 - intermittent water stress 2020 experiment.

**Additional file 5: Fig. S4.** Summary and physical locations of the QTL identified to be associated with root cone angle across the four experiments conducted, along with previously identified QTL in the literature (Bettembourg et al. 2017; Hanzawa et al. 2013; Kitomi et al. 2015; Lou et al. 2015; Uga et al. 2015; Uga et al. 2013a; Uga et al. 2013b). Physical map position are based on the Nipponbare sequence at RAP database. GH - Glasshouse Experiment; IWS19 - intermittent water stress 2019 experiment, WW20 - well-watered 2020 experiment, and IWS20 - intermittent water stress 2020 experiment.

## Data Availability

The datasets used and/or analysed during the current study are available from the corresponding author on reasonable request.

## Abbreviations

3KRGP: 3 K Rice Genome Project
AP: aerobic rice production
BLUP: best linear unbiased predictor
DArT: diversity arrays technology
DArTSeq: diversity arrays technology genotyping-by-sequencing platform
DAS: days after sowing
GH: glasshouse
IWS: intermittent water stress
KP: Kinandang Patong
LOD: logarithm of odds
MAF: minimum allele frequency
MET: multi-environment trial
QTL: quantitative trait loci
RCA: root cone angle
RDR: ratio of deep roots
RGA: root growth angle
RIL: recombinant inbred line
RSA: root system architecture
UQ: University of Queensland
WW: well-watered
