# Supplementary material for: Stable and Novel Quantitative Trait Loci (QTL) Confer Narrow Root Cone Angle in an Aerobic Rice (Oryza sativa L.) Production System
Source: Rice (N Y). 2021 Mar 7;14:28. doi: 10.1186/s12284-021-00471-2 (PMC7937586; doi:10.1186/s12284-021-00471-2)
Supplement: Supplementary file 1 — Additional file 1: Table S1. Summary of the number of detected polymorphic SNPs between Sherpa and IRAT109 and their average distances per chromosome. Numbers in parentheses are number of SNPs and average distance in cM using the final 1394 set of markers. [file 12284_2021_471_MOESM1_ESM.docx]

Table S1. Summary of the number of detected polymorphic SNPs between Sherpa and IRAT109 and their average distances per chromosome. Numbers in parentheses are number of SNPs and average distance in cM using the final 1,394 set of markers.

| Chromosome | Total length (cM) | No. of SNPs | Ave. Distance, kb (cM) |
| --- | --- | --- | --- |
| 1 | 179.6 | 446 (268) | 93.74 (0.7) |
| 2 | 157.7 | 153 (79) | 230.93 (2.0) |
| 3 | 153.3 | 193 (103) | 186.78 (1.5) |
| 4 | 122.9 | 207 (128) | 170.96 (1.0) |
| 5 | 114.9 | 208 (116) | 140.31 (1.0) |
| 6 | 123.8 | 300 (143) | 101.93 (0.9) |
| 7 | 111.7 | 261 (136) | 112.79 (0.8) |
| 8 | 110.7 | 228 (103) | 118.74 (1.1) |
| 9 | 84.7 | 106 (49) | 212.01 (1.8) |
| 10 | 79.1 | 116 (61) | 200.63 (1.3) |
| 11 | 110.8 | 193 (108) | 143.62 (1.0) |
| 12 | 111.3 | 213 (100) | 123.29 (1.1) |
