# Supplementary figures and images for: Stable and Novel Quantitative Trait Loci (QTL) Confer Narrow Root Cone Angle in an Aerobic Rice (Oryza sativa L.) Production System
Source: Rice (N Y). 2021 Mar 7;14:28. doi: 10.1186/s12284-021-00471-2 (PMC7937586; doi:10.1186/s12284-021-00471-2)

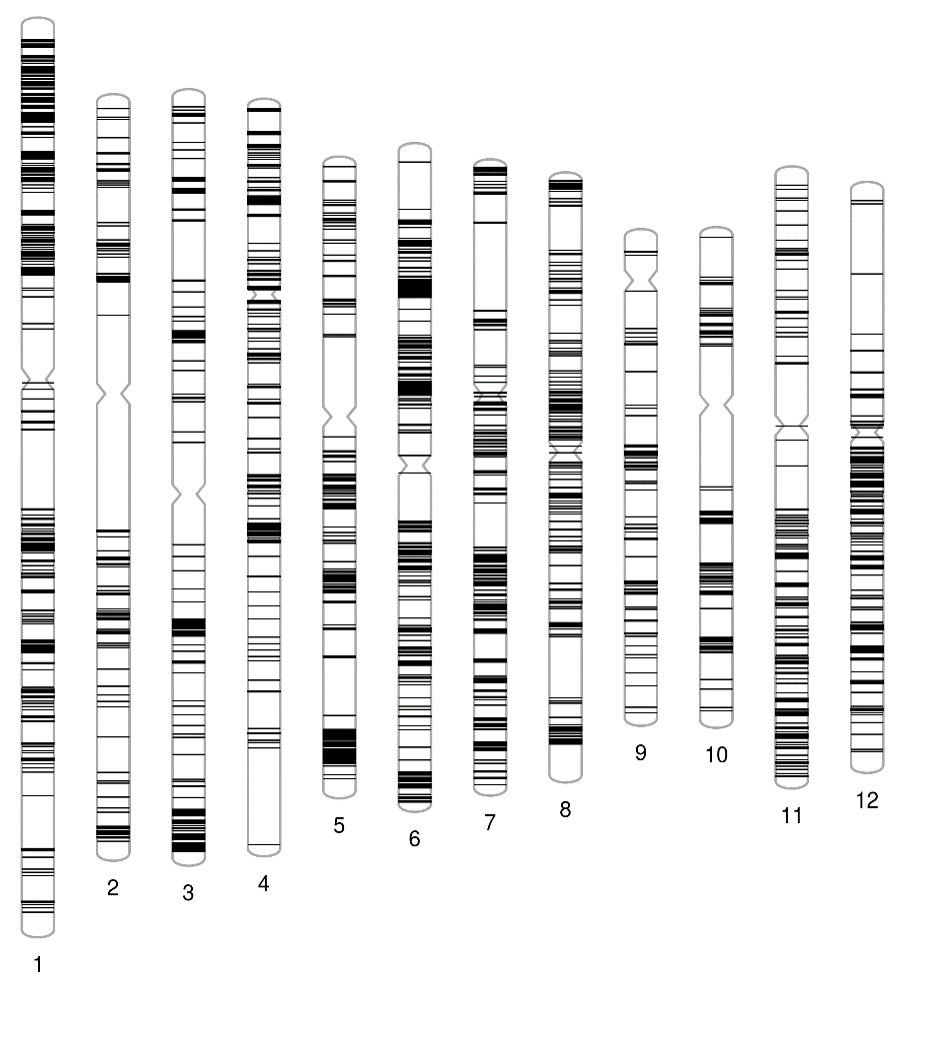

Supplement: Supplementary file 2 — Additional file 2: Fig. S1. Graphical genotype of the 2624 polymorphic markers detected between Sherpa and IRAT109 using DaRTSeq. [file 12284_2021_471_MOESM2_ESM.jpg]

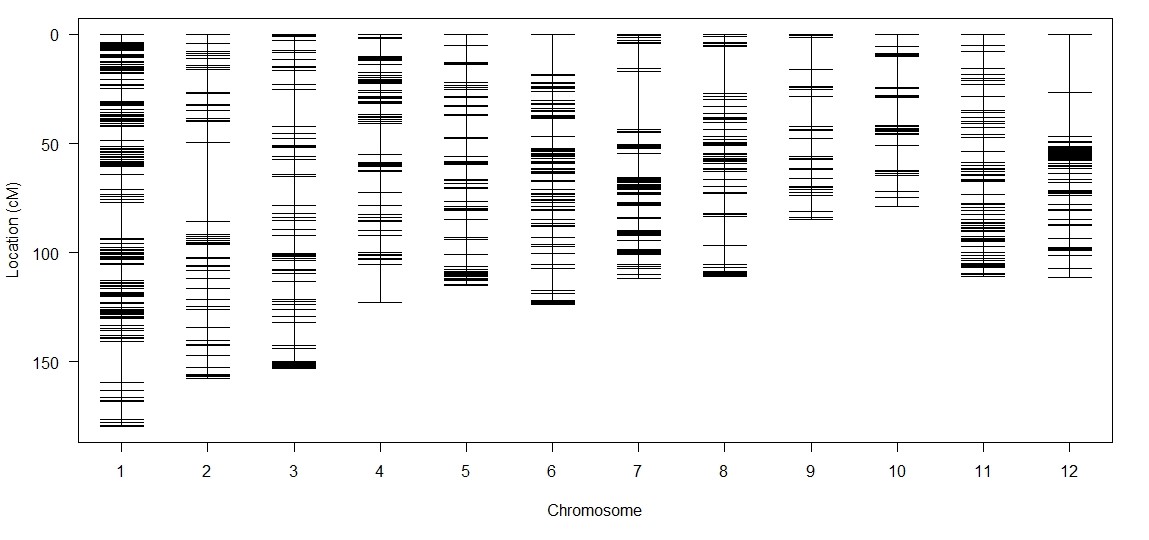

Supplement: Supplementary file 3 — Additional file 3: Fig. S2. Linkage map constructed from 1394 markers using RILs derived from Sherpa and IRAT109. (JPEG 101 kb) [file 12284_2021_471_MOESM3_ESM.jpeg]

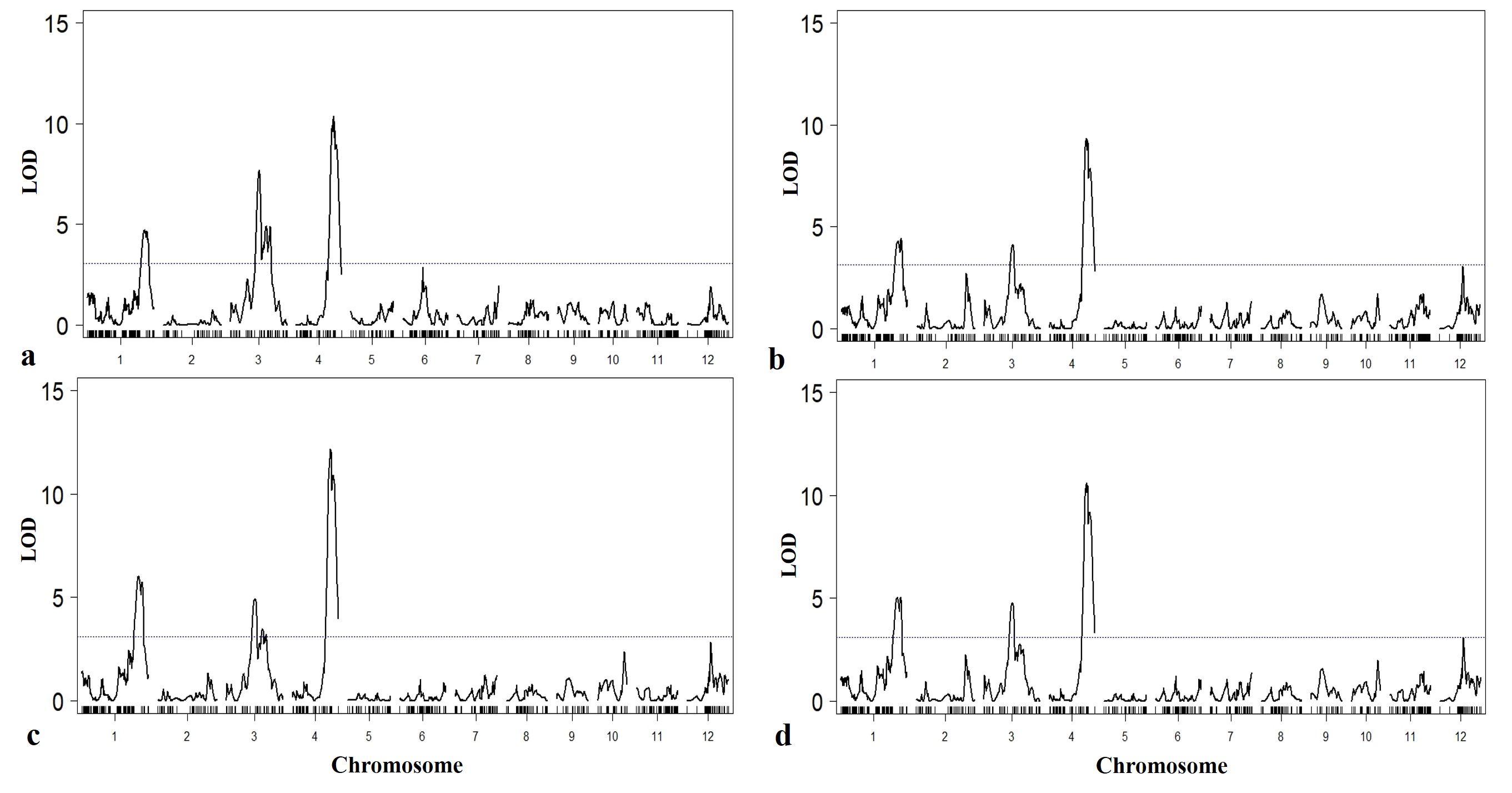

Supplement: Supplementary file 4 — Additional file 4: Fig. S3. LOD profiles for QTLs associated with root cone angle detected using single QTL model for (a) GH, (b) IWS19, (c) WW20, and (d) IWS20. Dotted blue line indicates LOD threshold. GH - Glasshouse Experiment; IWS19 - intermittent water stress 2019 experiment, WW20 - well-watered 2020 experiment, and IWS20 - intermittent water stress 2020 experiment. [file 12284_2021_471_MOESM4_ESM.jpg]

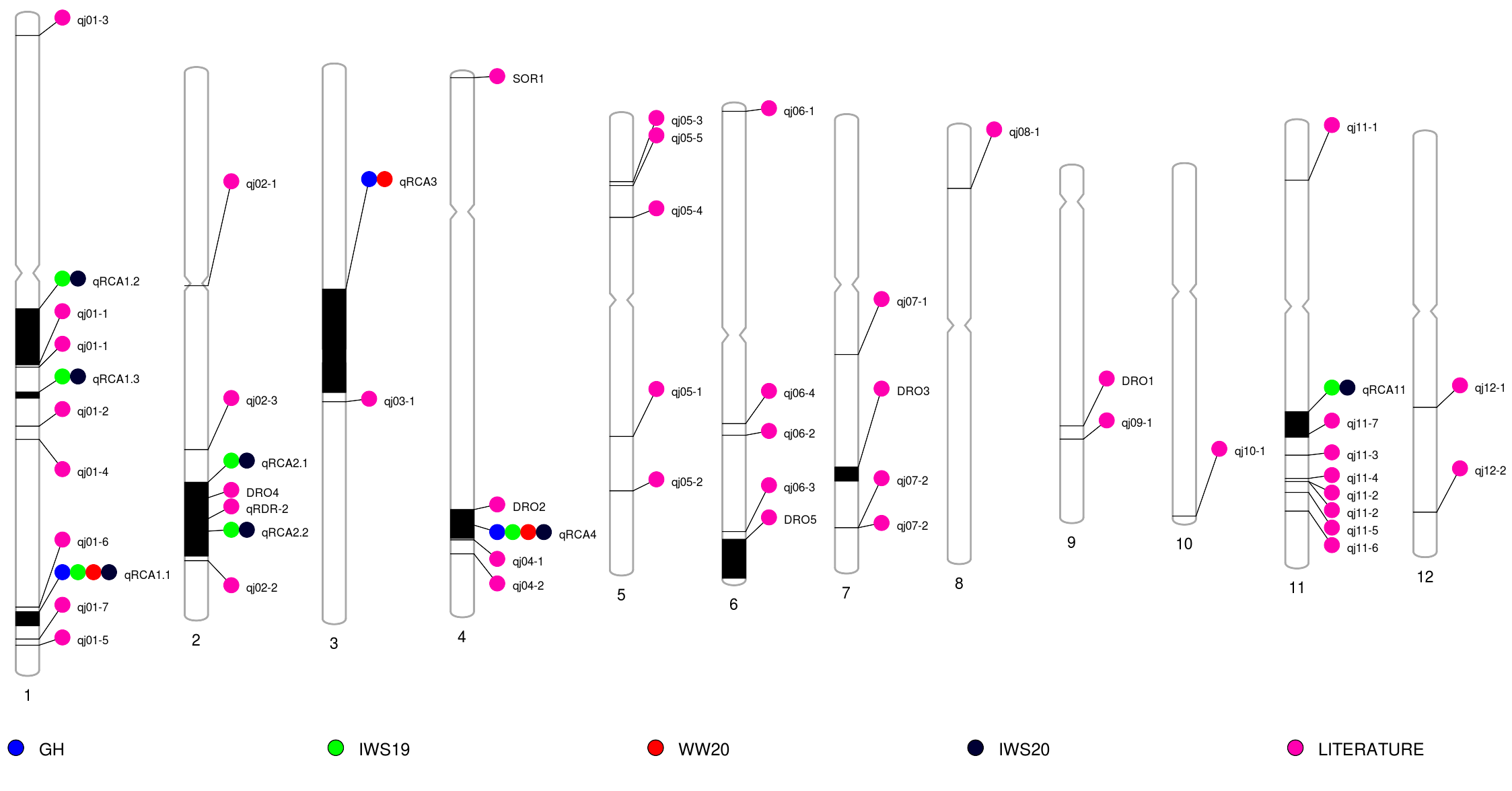

Supplement: Supplementary file 5 — Additional file 5: Fig. S4. Summary and physical locations of the QTL identified to be associated with root cone angle across the four experiments conducted, along with previously identified QTL in the literature (Bettembourg et al. 2017; Hanzawa et al. 2013; Kitomi et al. 2015; Lou et al. 2015; Uga et al. 2015; Uga et al. 2013a; Uga et al. 2013b). Physical map position are based on the Nipponbare sequence at RAP database. GH - Glasshouse Experiment; IWS19 - intermittent water stress 2019 experiment, WW20 - well-watered 2020 experiment, and IWS20 - intermittent water stress 2020 experiment. [file 12284_2021_471_MOESM5_ESM.png]
